# Transsynaptic complex dysfunction in the hippocampus of Alzheimer’s disease patients

**DOI:** 10.3389/fnagi.2026.1837327

**Published:** 2026-06-10

**Authors:** Ashly Hindle, Yong Chen, Xiangling Yin, Maria Manczak, Boris Decourt, Volker Neugebauer

**Affiliations:** 1 Garrison Institute on Aging, Texas Tech University Health Sciences Center, Lubbock, TX, United States; 2 Department of Pharmacology and Neuroscience, Texas Tech University Health Sciences Center, Lubbock, TX, United States; 3 Center of Excellence for Translational Neuroscience and Therapeutics, Texas Tech University Health Sciences Center, Lubbock, TX, United States; 4 Department of Neurology, Texas Tech University Health Sciences Center, Lubbock, TX, United States

**Keywords:** Alzheimer’s disease, autophagy, hippocampus, human brain, neuroplasticity, transsynaptic signaling complex

## Abstract

**Introduction:**

Alzheimer’s disease (AD) involves not only amyloid-β and tau pathology but synaptic dysfunction and impaired autophagy, though the underlying mechanisms and their relationship to AD progression are not well understood. Transsynaptic complexes involving presynaptic neurexins (Nrxn1/2/3), secreted cerebellins (Cbln1/2/3/4), and postsynaptic glutamate delta receptors (GluD1/2) play critical roles in organizing synapses and synaptic plasticity. Studies in pain models have reported that treatment with recombinant Cbln1 rescues AMPA glutamate receptor imbalance, promotes autophagy, and inhibits hyperexcitability and pain behaviors. Here we tested the novel hypothesis that dysregulation of Cbln-GluD-based transsynaptic complexes may occur in the brain of AD patients, providing insights into disease progression and potential avenues for therapeutic development.

**Methods:**

We analyzed human hippocampal tissues from the TTUHSC Garrison Brain Bank and the NIH NeuroBioBank for expression of transsynaptic complex components in addition to autophagy and neuroplasticity pathways. Their expression in hippocampus was compared between control samples of Braak stages 0/1 and AD samples showing either mild (Braak stage 2) or severe (Braak stages 5/6) neurofibrillary tangle pathology. Co-immunoprecipitation was used to examine protein–protein interactions.

**Results:**

We found significantly decreased protein levels of Cbln1 and GluD2 in AD hippocampus. In the autophagy pathway, PIST and beclin-1 were decreased in AD hippocampus. Co-immunoprecipitation revealed interactions between GluD1 and PIST and between PIST and beclin-1, suggesting possible regulatory interactions between transsynaptic complex elements and autophagy in human hippocampus. We further observed decreased BDNF, consistent with diminished neuroplasticity. Finally, cofilin phosphorylation was decreased in AD, suggesting disruption of trafficking and formation of cofilin-actin rods.

**Discussion:**

These results suggest that the homeostasis of signaling molecules important for synaptic integrity is disrupted in the human hippocampus at both early- and late-stage AD. The loss of transsynaptic complex expression is accompanied by the downregulation of autophagy and neuroplasticity markers that are known to be linked to AD pathology.

## 1 Introduction

Alzheimer’s disease (AD) is defined by neuropathologic changes centered on amyloid-β (Aβ) plaques (A), tau tangles (T), and neurodegeneration (N) (Jack et al., 2016). AD also involves neuroinflammation, abnormal autophagy, and diminished neuroplasticity (Parhizkar and Holtzman, 2022; Festa et al., 2021; Benarroch, 2018), and their interplay in AD pathology is not entirely clear. There is evidence to suggest that neural network function is impaired at early stages of AD, including in the hippocampus, and may precede clinically detectable ATN pathology and cognitive symptoms (Tang et al., 2023; Sperling et al., 2010; Cirrito et al., 2005). Given the lack of highly effective and widely available treatments, a better understanding of neuroplastic changes may offer new avenues for early interventions.

Tripartite transsynaptic complexes involving neurexins (Nrxn), cerebellins (Cbln), and delta-class glutamate receptors (GluD) play important roles in synapse formation and plasticity (Andrews and Dravid, 2021; Wennagel and Charrier, 2025). Cbln1, Cbln2, and Cbln4 are expressed in hippocampus and cerebral cortex, key brain regions affected in AD (Tao et al., 2018; Sudhof, 2023). Both GluD1 and GluD2 are expressed in hippocampus and cerebral cortex, although GluD1 is predominant (Nakamoto et al., 2020; Hepp et al., 2015). Certain Nrxn-Cbln-GluD combinations predominate in specific brain regions or types of synapses (Wennagel and Charrier, 2025). Relevant to this study, Nrxn1-Cbln2-GluD1 complexes are important for synapse formation and maintenance in the hippocampus (Tao et al., 2018). Nrxn1-Cbln2-GluD1 complexes are also important for NMDA receptor upregulation in the subiculum of the hippocampus, while Nrxn3-Cbln2-GluD1 complexes downregulate AMPA receptors (Sudhof, 2023). Although different combinations of these components can produce distinct effects, there may be a degree of functional overlap which could be exploited to achieve therapeutic responses outside of the brain regions and synapses of natural expression (Suzuki et al., 2020; Tao et al., 2018). For example, Cbln1, Cbln2, and Cbln4 are all able to increase AMPA and NMDA excitatory postsynaptic currents when expressed in CA1 pyramidal neurons even though these synapses natively employ Cbln2-mediated signaling through GluD1 (Tao et al., 2018).

GluD receptors are not traditional glutamate-gated ion channels like AMPA and NMDA receptors. Rather, their designation as glutamate receptors is based on sequence homology to subunits of AMPA, NMDA, and kainate receptors recognized at the time of discovery (Tricoire and Hepp, 2023; Piot et al., 2023). While cation permeability of GluD receptors has been reported (Kumar et al., 2023), the significance of these currents is debated (Itoh et al., 2024), and the most important roles of GluD1/2 are likely as transducers of non-ionotropic signals that regulate glutamate receptor density and autophagy (Fossati et al., 2019; Gawande et al., 2022). GluD1 and GluD2 have been found to interact with PIST (Yue et al., 2002; Narasimhan et al., 2025), and beclin-1 expression is significantly decreased in the somatosensory cortex of GluD1 conditional knockout mice (Gawande et al., 2022), further connecting the transsynaptic complex to autophagy machinery.

While Nrxn-Cbln-GluD transsynaptic complex dysfunction in AD remains to be explored, neurexin expression has been studied in J20 AD mice, which were found to express lower levels of hippocampal and cortical β-neurexins than wildtypes (Naito et al., 2017). Studies on Cblns in AD are similarly sparse. Cbln4 protected against Aβ-induced damage in cultured neurons from mouse hippocampus, likely by promoting GABAergic connections (Chacon et al., 2015), and a synthetic fusion protein of Cbln1 and neuronal pentraxin 1 improved symptoms in the 5xFAD mouse model of AD (Suzuki et al., 2020). The role of GluD receptors in AD is not known.

Recent research in chronic pain models characterized by changes in neuroplasticity and neuroimmune signaling has found that dysfunction of GluD1 signaling plays an important role in the abnormal neural excitability and autophagic turnover of AMPA receptors in a limbic region (amygdala), which can be mitigated with recombinant Cbln1 administration into the brain (Narasimhan et al., 2025). Building on this and other evidence from the literature described above, we hypothesized that dysregulation of Cbln-GluD complexes may occur in AD. There is currently a knowledge gap around whether transsynaptic complex dysfunction is a feature of human AD neuropathology. To avoid concerns about translatability from animal models, we used human brains to analyze Cbln, GluD, and markers of autophagy and neuroplasticity in hippocampal samples from control individuals and from AD patients showing either mild [Braak stage (BB) 2] or severe (BB5/6) tau neuropathology. To the best of our knowledge, the interaction of transsynaptic complex and autophagy machinery has not been studied in humans or in the context of AD. Our data suggest that a deficiency in Cbln-GluD transsynaptic signaling is associated with altered autophagy, neuroplasticity, and actin-mediated trafficking in the hippocampus of AD patients. Future studies are needed to define how these pathways functionally relate to each other under conditions of normal and pathological hippocampal physiology.

## 2 Methods

### 2.1 Subjects and samples

Human hippocampal samples were obtained from the Texas Tech University Health Sciences Center (TTUHSC) Garrison Brain Bank (GBB) and the National Institutes of Health (NIH) NeuroBioBank (NBB) (Freund et al., 2018). Three study groups were defined according to Braak staging, a postmortem measure of tau pathology: Controls (BB0/1), AD with mild tau pathology (BB2), and AD with severe tau pathology (BB5/6).

#### 2.1.1 GBB samples

Hippocampal tissues from control individuals (BB0/1, *n* = 5) and AD patients of mild (BB2, *n* = 3) or severe (BB5/6, *n* = 6) tau pathology were used for protein studies. All subjects were female from real-world settings, i.e., none of the subjects were part of a clinical observational study on dementia before death, making this cohort unique for investigations on dementia-related molecular mechanisms. Tissues were donated to the TTUHSC Garrison Institute on Aging with informed consent of next of kin. Research using postmortem human specimens does not require institutional review board approval under Federal Regulation 45 CFR 46. Demographic and pathological information is presented in Supplementary Table S1. Right hemispheres were fixed in 10% buffered formalin and examined by licensed neuropathologists. Supplementary Figure S1 shows representative examples of pTau staining using the AT8 antibody from one control hippocampus, one AD hippocampus of BB2, and one AD hippocampus of BB5/6 performed by Tissue Techniques Pathology Labs, Inc. (Dallas, TX, USA) for use by the neuropathologist. Left hemispheres were sectioned coronally at 1 cm, vacuum sealed, and stored at −80 °C. The quality of banked tissue was validated using a 2,100 Bioanalyzer (Agilent Technologies, Santa Clara, CA, USA). All specimens had an RNA Integrity Number (RIN) of 4 or above, and there were no significant differences in RIN between groups.

#### 2.1.2 NBB samples

Hippocampal tissue from control individuals (BB0/1, *n* = 5) and AD patients of mild (BB2, *n* = 9), or severe (BB5/6, *n* = 9) tau pathology were utilized for qPCR experiments. Demographic and pathological information shown in Supplementary Table S2 was supplied by the NIH NBB. Tissues were obtained under NBB request ID#1455 and Federal Wide Assurance number FWA00006767.

### 2.2 Western blot

Tissues were lysed in RIPA buffer (cat#89901, Thermo Fisher, Waltham, MA, USA) with cOmplete Mini Protease Inhibitor Cocktail (cat#11836153001, MilliporeSigma, Burlington, MA, USA) or in Pierce IP lysis buffer (cat#87787, Thermo Fisher) containing Halt Protease and Phosphatase Inhibitor (cat#78440, Thermo Fisher). Protein concentration was measured using Pierce BCA assay (cat#A55865, Thermo Fisher). Protein was prepared for gel electrophoresis in LDS buffer containing β-mercaptoethanol. Equal amounts of total protein were loaded onto SDS-PAGE gels, transferred to PVDF membranes, and blocked in TBST (TBS and 0.1% Tween 20) containing 5% w/v fat-free dry milk for 2 h. The membranes were then incubated with primary rabbit antibodies: anti-Cbln1 (1:500; cat#PA5-101513, Thermo Fisher), anti-Cbln2 (1:1000; cat#PA5-36471, Thermo Fisher), anti-GluD1 (1:1000; cat#13040-1-AP, Proteintech, Rosemont, IL, USA), anti-GluD2 (1:1000; cat#AGC039, Alomone Labs, Jerusalem, Israel), anti-beclin-1 (1:1000; cat#PA1-16857, Thermo Fisher), anti-PIST (GOPC, cat#12163-1-AP, Proteintech), anti-BDNF (1:500; cat#PA5-85730, Thermo Fisher), anti-phosphorylated cofilin (1:1000; cat#3313, Cell Signaling, Danvers, MA, USA), and anti-cofilin (1:1000; cat#3318, Cell Signaling) overnight at 4 °C. The PVDF membranes were then washed 3 times with TBST and incubated with anti-rabbit peroxidaseconjugated secondary antibody (cat#1706515, Bio-Rad Laboratories, Hercules, CA, USA) for 1 h at room temperature. Proteins were detected using SuperSignal™ West Femto Maximum Sensitivity Substrate (cat#34094, Thermo Fisher) or Pierce ECL (cat#32106, Thermo Fisher) and imaged using an Azure c400 imager (Azure Biosystems, Dublin, CA, USA). For the detection of total cofilin expression, the p-cofilin blots were stripped with Restore™ PLUS Western Blot Stripping Buffer (cat#46430, Thermo Fisher) and re-probed with anti-cofilin. The β-actin housekeeping gene served as a loading control and was probed using a primary mouse antibody (cat#MA1-140, Thermo Fisher) followed by anti-mouse peroxidase-conjugated secondary (cat#1706516, Bio-Rad). Band intensities were quantified using ImageJ software and normalized to β-actin (cat#MA1-140, Thermo Fisher). The rectangle tool in ImageJ was used to manually box each band for densitometry. Boxes were equally sized and non-overlapping, with boundaries between lanes.

### 2.3 Co-immunoprecipitation

Protein G Dynabeads (cat#10007D, Thermo Fisher) were incubated with 10 μg of anti-PIST antibody (cat#sc393026, Santa Cruz, Dallas, TX, USA) or anti-beclin-1 antibody (cat#sc-48341, Santa Cruz) in PBS overnight at 4 °C with gentle rotation. Antibody-bead complexes were crosslinked using 2 mM DSS (disuccinimidyl suberate; Thermo Fisher) in PBS for 30 min at room temperature. The reaction was quenched with 50 mM Tris–HCl (pH 7.5) for 15 min. Beads were washed 3 times with Pierce IP lysis buffer (cat#87787, Thermo Fisher) to remove unreacted crosslinker. Crosslinked beads were incubated overnight at 4 °C with pooled lysates from control hippocampus or AD hippocampus (1,000 μg total protein) with gentle rotation. Following washing with lysis buffer, bound proteins were eluted using 0.1 M glycine (pH 2.8), neutralized with 1 M Tris–HCl (pH 8.0), and analyzed by SDS-PAGE and immunoblotting with antibodies targeting PIST (GOPC, cat#12163-1-AP, Proteintech), beclin-1 (cat#66665-1-Ig, Proteintech) and GluD1 (cat#13040-1-AP, Proteintech) followed by appropriate HRP-conjugated secondary antibodies. Protein bands were visualized using Pierce ECL (cat#32106, Thermo Fisher).

### 2.4 Real-time PCR

RNA was extracted from NBB samples using RNAzol RT (cat#RN190, Molecular Research Center, Inc. Cincinnati, OH). RNA concentration was assessed by NanoDrop 2000 (Thermo Fisher). cDNA was synthesized using the Maxima First Strand cDNA Synthesis Kit (cat#K1642, Thermo Fisher). Real-time PCR was conducted using the QuantStudio 7 RT-PCR system (Thermo Fisher) using PowerUp SYBR Green Master Mix (cat#A25742, Thermo Fisher). Primer sequences are shown in Supplementary Table S3. Relative expression was calculated using the 2^−ΔΔCt^ methodology, normalized to *ACTB* (β-actin), and statistical comparisons employed ΔΔCt values.

### 2.5 Statistics

Statistical analysis was performed using GraphPad Prism version 8.0 (GraphPad Software Inc., Boston, MA, USA) or calculated manually in Microsoft Excel using Prism outputs. The Shapiro–Wilk test was used to test for normality, and the Brown–Forsythe test was used to evaluate homoscedasticity. One-way ANOVA with Tukey’s multiple comparisons test was used to analyze data meeting the conditions of normality and homoscedasticity. Data that did not meet these conditions were analyzed by the nonparametric Kruskal–Wallis test with Dunn’s multiple comparisons test. Eta-squared (*η^2^*, labeled as R squared in Prism 8.0 output) was used as a measure of effect size for ANOVA analysis. The absolute value of Cohen’s *d* (presented simply as *d*) was used as a measure of effect size magnitude for Tukey’s multiple comparisons test.

$$d=\frac{M_{1}-M_{2}}{\surd {\mathrm{MS}}_{\text{residual}}}$$

Where *M*_1_ is the mean of group 1, *M*_2_ is the mean of group 2, and MS_residual_ is the residual mean square provided by Prism 8.0 output. As a measure of effect size following the Kruskal–Wallis test we calculated eta-squared from the Kruskal-Wallis *H* statistic (*η*^2^*_H_*).

$$n_{H}^{2}=(H-k+1)/(n_{\text{total}}-k)$$

Where *H* is the Kruskal–Wallis statistic provided by Prism 8.0 output, *k* is the number of groups, and *n*_total_ is the total number of samples across all groups. The rank-correlation coefficient (*r*) is present as a measure of effect size following Dunn’s multiple comparisons test.

$$r=|z|/\surd (n_{\text{total}})$$

Where *z* is the standardized *z*-test statistic provided by Prism 8.0 output and *n*_total_ is the total number of samples in the two groups being compared. Bar graphs depict the mean ± SEM. *p-*values less than 0.05 were considered significant. Protein and RNA expression of each AD group was determined relative to the mean of the control group which was defined as equal to 1.

## 3 Results

### 3.1 Decreased protein levels of transsynaptic complex components in AD hippocampus

**Figure 1:**
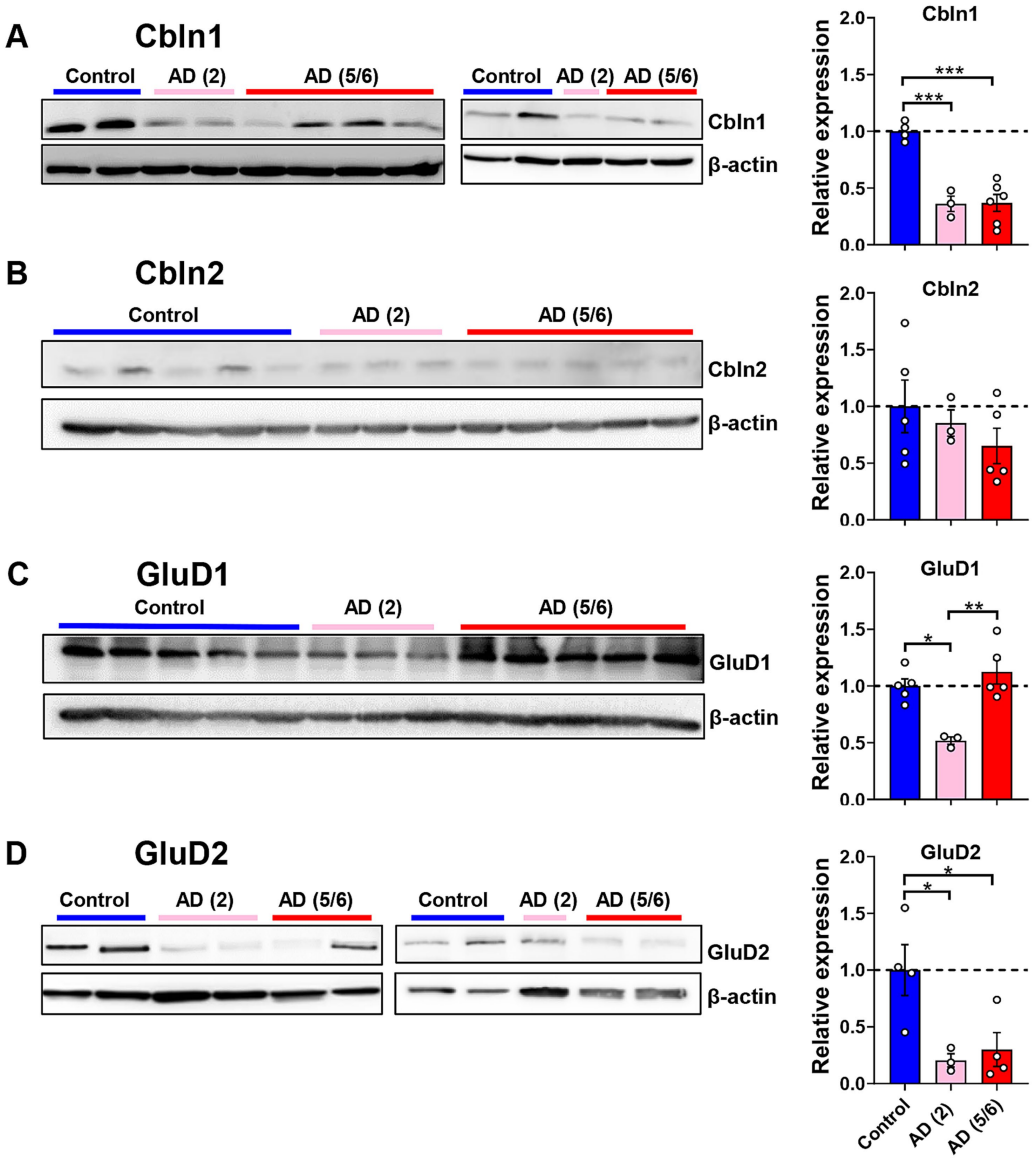
Transsynaptic complex proteins are decreased in AD hippocampus. Protein levels of Cbln1/2 and GluD1/2 transsynaptic complex proteins were compared between control (blue) and AD hippocampus of Braak stages 2 (BB2, pink) or 5/6 (BB5/6, red) using the GBB sample set. (**A**) Cbln1 showed significant downregulation in AD hippocampus of BB2 and BB5/6 compared to control samples. (**B**) Cbln2 was not significantly different. (**C**) GluD1 was significantly decreased only in AD hippocampus of BB2, whereas (**D**) GluD2 was significantly decreased relative to controls in both BB2 and BB5/6 AD hippocampus. Cbln1 (**A**) and GluD2 (**D**) were analyzed using two separate western blots with relative expression in AD hippocampus determined relative to controls on the same blot. Data were analyzed by one-way ANOVA with Tukey’s test. ^*^p < 0.05; ^**^p < 0.01; ^***^p < 0.001.

To examine whether transsynaptic complex components are disrupted in the hippocampus of AD patients, we first examined the protein levels of Cbln1/2 and GluD1/2 in the GBB sample set. There was a significant effect of experimental group on the hippocampal protein level Cbln1 (*p* = 0.0001, *η*^2^ = 0.839), but this was not observed for Cbln2 (*p* = 0.4242, *η*^2^ = 0.158). Cbln1 was significantly decreased in AD hippocampus of both BB2 (*p* = 0.0005, *d* = 4.38) and BB5/6 (*p* = 0.0001, *d* = 4.33) compared to controls of BB0/1 (Figure 1A). In contrast, Cbln2 was not significantly different from controls in AD hippocampus of BB2 (*p* = 0.8747, *d* = 0.36) or BB5/6 (*p* = 0.3947, *d* = 0.86), but there was a trend toward decrease as Braak stage increased (Figure 1B). We observed significant effects of experimental group on the protein levels of both GluD1 (*p* = 0.0026, *η*^2^ = 0.697) and GluD2 (*p* = 0.0224, *η*^2^ = 0.613). GluD1 was decreased in BB2 AD hippocampus relative to controls (*p* = 0.0101, *d* = 2.72) and BB5/6 AD hippocampus (*p* = 0.0023, *d* = 3.42) but was not decreased in BB5/6 AD hippocampus relative to controls (*p* = 0.5284, *d* = 0.70, Figure 1C). GluD2 was significantly decreased in both BB2 (*p* = 0.0340, *d* = 2.38) and BB5/6 (*p* = 0.0426, *d* = 2.10) AD hippocampus relative to controls (Figure 1D). These results support protein-level decreases of transsynaptic complex components in AD.

### 3.2 Decreased expression of key mediators of autophagy in AD hippocampus

**Figure 2:**
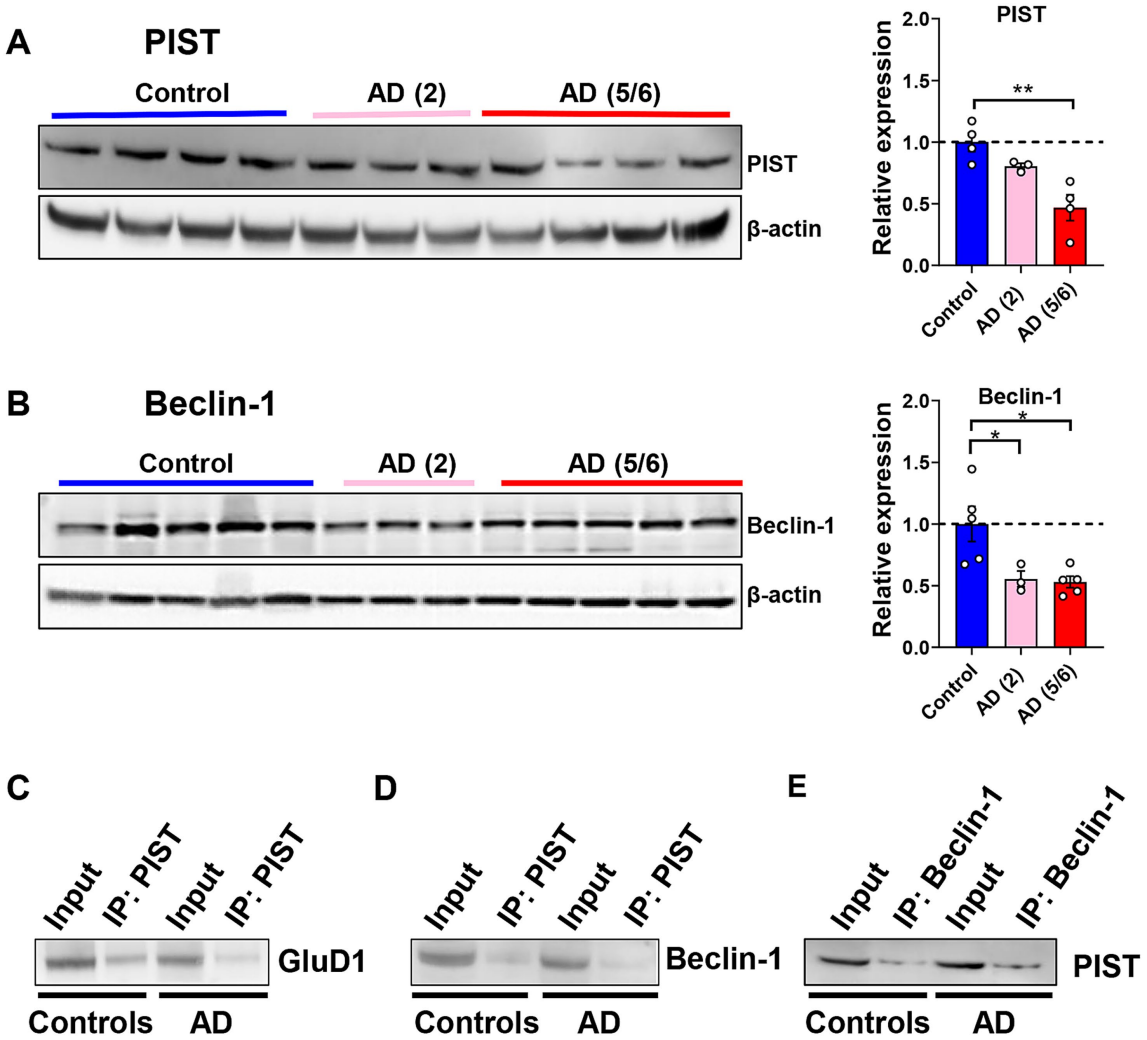
Dysregulation of autophagy in AD hippocampus. Expression levels were compared between control (blue) and AD hippocampus of Braak stages 2 (BB2, pink) or 5/6 (BB5/6, red) using the GBB sample set. **(A)** PIST was significantly decreased in AD hippocampus at BB5/6 relative to control samples. β-actin corresponding to PIST was obtained from a separate blot using the same samples. **(B)** Beclin-1 was significantly decreased in AD hippocampus of both BB2 and BB5/6. **(C–E)** GluD1 interacts with autophagy machinery**. (C)** Co-immunoprecipitation was used to investigate the possible interaction between GluD1 and PIST in pooled AD hippocampus samples and pooled control samples. GluD1 protein was detected following immunoprecipitation (IP) by anti-PIST antibody, connecting transsynaptic complexes to autophagy machinery. **(D)** Beclin-1 was detected following immunoprecipitation by anti-PIST antibody, and **(E)** PIST was detected following immunoprecipitation with anti-Beclin-1 antibody. Data were analyzed by one-way ANOVA with Tukey’s multiple comparisons test. ^*^p < 0.05; ^**^p < 0.01.

We then examined protein expression of PIST and beclin-1 in the GBB sample set. These proteins are regulators of autophagy that have been reported to function downstream of GluD1/2 (Yue et al., 2002; Narasimhan et al., 2025). Notably, GluD1, the predominant GluD in hippocampus, has been shown to interact with PIST in mice (Narasimhan et al., 2025). There were significant effects of experimental group on the hippocampal protein levels of both PIST (*p* = 0.0047, *η*^2^ = 0.738) and beclin-1 (*p* = 0.0127, *η*^2^ = 0.583). We found a significant decrease in PIST relative to controls in AD hippocampus of BB5/6 (*p* = 0.0039, *d* = 3.33) but not BB2 (*p* = 0.2992, *d* = 1.22 Figure 2A). Beclin-1 protein was significantly decreased compared to controls in AD hippocampus of both BB2 (*p* = 0.0443, *d* = 2.06) and BB5/6 (*p* = 0.0161, *d* = 2.17, Figure 2B). These results support a decrease of key regulators of autophagy in AD hippocampus.

### 3.3 Interaction between GluD1 and PIST in control and AD hippocampus

Interaction of GluD1 and PIST has been reported in mice (Yue et al., 2002; Narasimhan et al., 2025), but to our knowledge this has not been studied in human neurons or hippocampus. We therefore performed a co-immunoprecipitation experiment using pooled human hippocampus tissue to examine interactions between GluD1 and PIST and between PIST and beclin-1. We detected an interaction between GluD1 and PIST (Figure 2C). Additionally, beclin-1 was found to co-precipitate with PIST (Figure 2D) and vice versa (Figure 2E). This GluD1-PIST-beclin-1 axis of interaction suggests that autophagy may be regulated by GluD1-utilizing transsynaptic complexes in human hippocampus under normal physiological conditions and under AD-pathological conditions.

### 3.4 Decreased synaptic plasticity marker BDNF in AD hippocampus

**Figure 3:**
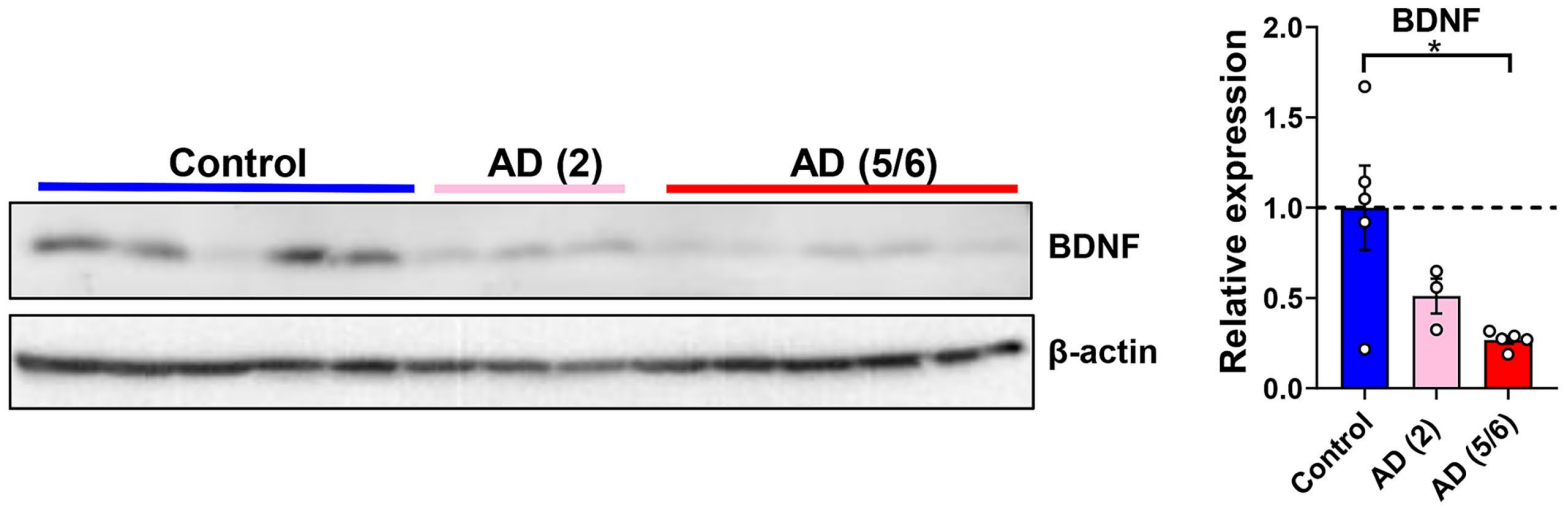
BDNF is decreased in AD hippocampus. BDNF is a key regulator of synaptic plasticity which induces downstream changes in the abundance of ligand-gated ion channels which determine neuronal excitability. Levels of BDNF were compared between control (blue) and AD hippocampus of Braak stages 2 (BB2, pink) or 5/6 (BB5/6, red) using the GBB sample set. BDNF was significantly decreased in AD hippocampus of BB5/6 relative to controls. Data were analyzed by one-way ANOVA with Tukey’s multiple comparisons test. ^*^p < 0.05.

Brain-derived neurotrophic factor (BDNF), an important player in synaptic plasticity, can regulate abundance and function of ligand gated ion channels and modulate neuronal excitability (Wan et al., 2024; Caldeira et al., 2007). In AD patients, decreased brain and serum BDNF are associated with faster cognitive decline (Laske et al., 2011; Buchman et al., 2016). In mice, it was shown that exercise-induced improvements in learning and memory were mediated by bi-directional positive feedback between BDNF and autophagy in the hippocampus (Khoury et al., 2023). Using the GBB sample set, we found a significant effect of experimental group on the hippocampal protein level of BDNF (*p* = 0.0201, *η*^2^ = 0.542). Relative to controls we observed decreases in BDNF that were significant in BB5/6 AD hippocampus (*p* = 0.0170, *d* = 2.15) but did not reach significance in BB2 AD hippocampus (*p* = 0.1705, *d* = 1.44, Figure 3). This supports a progressive deficiency in the BNDF-dependent regulation of hippocampal synaptic plasticity and autophagy in the AD cases.

### 3.5 Dysregulation of cofilin phosphorylation in AD hippocampus

Cofilin is a regulator of actin dynamics that depolymerizes actin filaments, and active regulation of cofilin phosphorylation impacts trafficking of AMPA receptors, making it important for synaptic plasticity (Gu et al., 2010; Sumi et al., 1999). GluD1 knockout mice have deceased levels of p-cofilin and altered ratios of NMDA subunits, connecting GluD1 signaling to the regulation of cofilin and plasticity (Gupta et al., 2015). Excessive dephosphorylation of cofilin has been found to disrupt microtubule dynamics as well as cause aggregation with actin into disruptive cofilin-actin rods in neuronal cultures from fetal rat brain, and cofilin aggregates were co-localized with Aβ plaques in human postmortem hippocampal sections (Minamide et al., 2000). The formation of cofilin-actin rods was also induced by synthetic oligomers of human Aβ_1-42_ in cultured hippocampal neurons and in slices from rat brain (Davis et al., 2011). Cofilin-actin rod formation was found to block intracellular trafficking and cause synaptic loss in cultured rat hippocampal neurons (Cichon et al., 2012). Interestingly, hippocampal tau pathology was increased in Tau-P301S;cofilin+/− mice transduced with constitutively dephosphorylated (active) cofilin, but not phosphomimetic (inactive) cofilin via AAV9 (Woo et al., 2019). Finally, work in human pulmonary endothelial cells has found that beclin-1 also promotes cofilin phosphorylation, connecting autophagy and cofilin regulation (Leonard et al., 2019).

**Figure 4:**
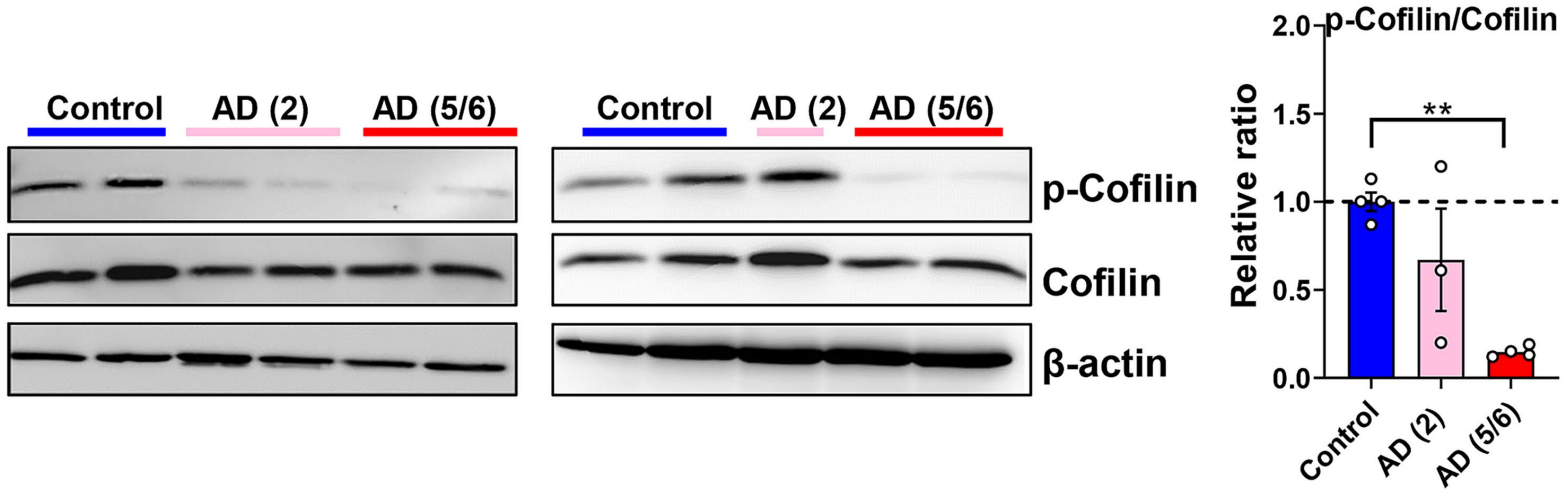
Dysregulation of cofilin in AD. Cofilin phosphorylation regulates actin dynamics, which affects AMPA receptor trafficking and synaptic plasticity, among other functions. Levels of cofilin and phosphorylated cofilin (p-cofilin) were compared between control (blue) and AD hippocampus of Braak stages 2 (BB2, pink) or 5/6 (BB5/6, red) using the GBB sample set. The p-cofilin/cofilin ratio was decreased in AD hippocampus of BB5/6 compared to controls. Cofilin and p-cofilin were analyzed using two separate western blots with relative expression in AD hippocampus determined relative to controls on the same blot. Data were analyzed by one-way ANOVA with Tukey’s multiple comparisons test. ^**^p < 0.01.

Given the connections of cofilin dynamics to GluD1, autophagy, plasticity, and AD pathology, we examined total cofilin and phospho-cofilin (p-cofilin) levels (Figure 4). We observed a significant effect of experimental group on the hippocampal ratio of p-cofilin to cofilin (*p* = 0.0052, *η*^2^ = 0.731). Samples from BB5/6 AD hippocampus showed significantly decreased p-cofilin/cofilin ratios relative to controls (*p* = 0.0042, *d* = 3.27) while BB2 AD hippocampus showed a non-significant trend toward decrease (*p* = 0.2775, *d* = 1.27, Figure 4). Thus, the observed changes in transsynaptic complex, autophagy, and plasticity pathways (Figures 1–3) were associated with deficient cofilin phosphorylation.

### 3.6 GluD2 mRNA is downregulated in AD hippocampus of advanced Braak stage

**Figure 5:**
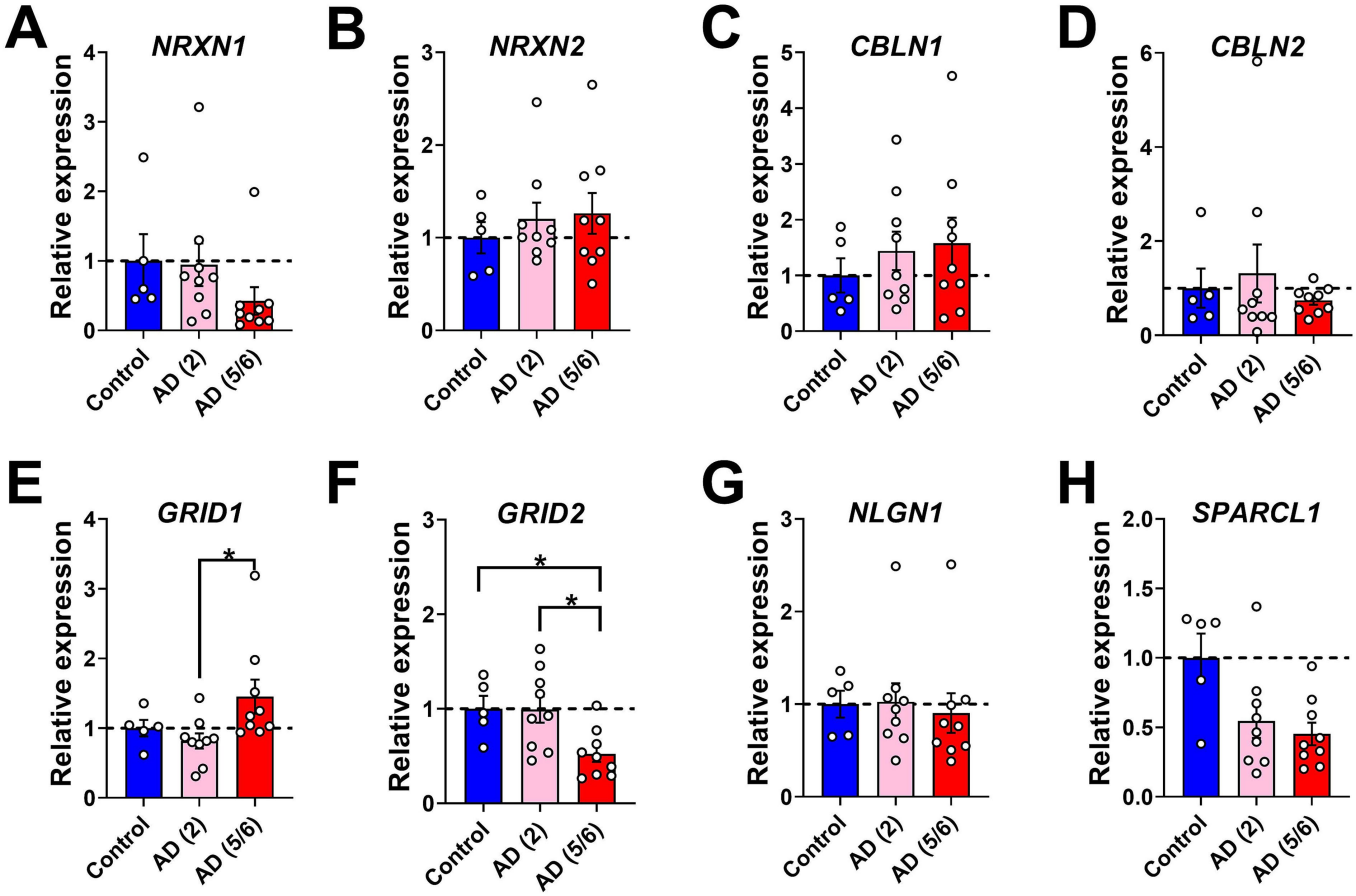
Transsynaptic complex mRNA expression. **(A–F)** mRNA transcripts encoding components of Nrxn-Cbln-GluD transsynaptic complexes were compared by qPCR between control (blue) and AD hippocampus of Braak stages 2 (BB2, pink) or 5/6 (BB5/6, red) using the sample set obtained from the NIH NeuroBioBank program. These included **(A)** *NRXN1*, **(B)** *NRXN2*, **(C)** *CBLN1*, **(D)** *CBLN2*, **(E)** *GRID1* (encoding GluD1), and **(F)** *GRID2* (encoding GluD2). *GRID1* mRNA was increased in BB5/6 AD hippocampus relative to BB2, and *GRID2* mRNA was decreased in AD hippocampus of BB5/6 relative to BB2 and control hippocampus. **(G,H)** Relative expression of mRNAs encoding components of the Nrxn-Nlgn transsynaptic complex, including *NLGN1* and *SPARCL1* (encoding hevin), an astrocytic protein that modulates Nrxn-Nlgn complex formation. Neither *NLGN1* or *SPARCL1* were significantly different in AD hippocampus relative to controls. **(A–G)** Data were analyzed by one-way ANOVA with Tukey’s multiple comparisons test. **(H)** Data were analyzed by Kruskal–Wallis test with Dunn’s multiple comparisons test. ^*^p < 0.05.

Finally, using samples from the NIH NeuroBioBank we compared mRNA levels of transsynaptic complex components between control hippocampus (*n* = 5) and AD hippocampus of BB2 (*n* = 9) or BB5/6 (*n* = 9) (Figure 5). Targets included *NRXN1/2*, *CBLN1/2*, and *GRID1/2* (GluD1/2) as well as *NLGN1*, encoding neuroligin-1, which forms an alternative transsynaptic complex with Nrxn1α, and *SPARCL1,* encoding the secreted astrocytic protein hevin, which bridges and modulates Nrxn1α-neuroligin interactions.

There was no significant effect of experimental group on hippocampal mRNA expression of *NRXN1* (*p* = 0.0509, *η*^2^ = 0.258), *NRXN2* (*p* = 0.7091, *η*^2^ = 0.034), *CBLN1* (*p* = 0.7507, *η*^2^ = 0.028), or *CBLN2* (*p* = 0.959, *η*^2^ = 0.004). Consistently, relative to controls there were no significant changes in mRNA in AD hippocampus of either BB2 or BB5/6 for *NRXN1* (*p* = 0.9250, *d* = 0.21 and *p* = 0.0888, *d* = 1.25, respectively, Figure 5A), *NRXN2* (*p* = 0.7314, *d* = 0.42 and *p* = 0.7335, *d* = 0.42, Figure 5B), *CBLN1* (*p* = 0.7561, *d* = 0.40 and *p* = 0.7892, *d* = 0.37, Figure 5C) or *CBLN2* (*p* = 0.9549, *d* = 0.16 and *p* = 0.9811, *d* = 0.10, Figure 5D). We observed significant effects of experimental group on hippocampal mRNA expression of *GRID1* (*p* = 0.0249, *η*^2^ = 0.309) and *GRID2* (*p* = 0.0069, *η*^2^ = 0.392). *GRID1* mRNA was significantly increased in BB5/6 AD hippocampus relative to BB2 AD hippocampus (*p* = 0.0192, *d* = 1.41), but there was no difference from controls in AD hippocampus of BB2 (*p* = 0.5088, *d* = 0.63) or BB5/6 (*p* = 0.3620, *d* = 0.78, Figure 5E). In contrast, *GRID2* mRNA was significantly downregulated in BB5/6 AD hippocampus relative to controls and BB2 AD hippocampus (*p* = 0.0237, *d* = 1.61 and *p* = 0.0133, *d* = 1.49) while there was no difference between controls and BB2 AD hippocampus (*p* = 0.9730, *d* = 0.12, Figure 5F). There was no significant effect of experimental group on *NLGN1* mRNA (*p* = 0.6957, *η*^2^ = 0.036), and *NLGN1* mRNA showed no significant changes relative to controls in AD hippocampus of BB2 (*p* = 0.9822, *d* = 0.10) or BB5/6 (*p* = 0.7313, *d* = 0.42, Figure 5G). Finally, there was no significant difference in *SPARCL1* mRNA expression across experimental groups (*p* < 0.0729, *η*^2^_H_ = 0.16). *SPARCL1* did not differ significantly from controls in either BB5/6 AD hippocampus (*p* = 0.768, *r* = 0.60) or BB2 AD hippocampus (*p* = 0.2058, *r* = 0.49), although a trend toward decrease was noted (Figure 5H). These results, coupled with the protein expression analyses, support a model in which decreases in hippocampal cerebellin levels in AD are likely due to protein-level interactions, while changes in hippocampal delta-class glutamate receptors could reflect both protein-level interactions and transcriptional regulation.

## 4 Discussion

In this work, we analyzed hippocampal tissues obtained from the Garrison Brain Bank and the NIH NeuroBioBank to compare protein and mRNA expression of transsynaptic complexes and proteins associated with autophagy and neuroplasticity between AD patients of BB2, AD patients of BB5/6, and non-AD control individuals. Our protein results suggest that the homeostasis of important proteins involved in synaptic integrity, autophagy, and neuroplasticity are disrupted in AD. In addition, disruptions to cofilin phosphorylation likely reflect impairment of the intracellular trafficking required for neuroplasticity and functional autophagy.

Our data provide a proof of principle that dysregulation of transsynaptic complexes is observed in human AD hippocampus. We found decreased expression of transsynaptic complex proteins and other proteins that directly or indirectly regulate neuroplasticity and autophagy, however it is presently unclear whether such disruption is a cause or consequence of AD pathology.

Our results are consistent with findings from previous studies on AD, including decreased BDNF (Buchman et al., 2016), Beclin-1 (Pickford et al., 2008), and p-cofilin in AD (Bamburg et al., 2010), conveying validity to the analysis of our human tissue samples. To our knowledge this is the first work reporting deficiencies in components of transsynaptic complexes in AD hippocampal tissues from human subjects. The downregulation of PIST in AD as well as the co-precipitation of PIST and GluD1 in human hippocampus, suggesting the possible regulation of hippocampal autophagy by the transsynaptic complexes, are also novel findings from our work, although additional studies are needed to clarify regulatory relationships.

In contrast to our protein results, our qPCR experiments showed few significant changes in mRNAs encoding transsynaptic complex components. While the inadequate clearance of Aβ and ptau in AD creates bottlenecks in the degradation of other proteins (Thibaudeau et al., 2018; Drummond et al., 2020), synaptic proteins are often decreased in AD due to synapse degeneration induced by soluble Aβ oligomers or impaired trafficking (Wilcox et al., 2011; Perdigao et al., 2020; Zhu et al., 2026). These latter phenomena, along with the observed differences between the relative levels of proteins and mRNAs, support a model where loss of transsynaptic complex proteins is driven mainly by proteinopathy rather than downregulation of mRNA.

Pathological loss of synapses is a feature of AD and strongly correlates with cognitive decline (Mecca et al., 2022; Long and Holtzman, 2019). Cbln1 protein has synaptogenic activity (Yuzaki, 2011), and, accordingly, we found that hippocampus from AD patients was deficient in Cbln1 as well as GluD1/2 relative to controls. This suggests that boosting Cbln or GluD components of transsynaptic complexes may counteract synaptic loss in AD. While we did not examine expression of Cbln1/2 or GluD1/2 in a cell-type specific manner, there is scant evidence for their expression in glial cell types. The observed protein-level decreases are therefore likely neuronal effects. Of the transsynaptic complex components studied, the greatest deficiencies were observed in Cbln1 and GluD2 suggesting that interventions to restore levels of these proteins may prove most beneficial.

Evidence suggests that interventions to strengthen transsynaptic connections, using Cbln1 or a fusion protein containing parts of Cbln1 and pentraxin-1 (CPTX), could beneficially regulate AMPA receptor levels in various disease models (Suzuki et al., 2020; Narasimhan et al., 2025). GluD1 is not only involved in synapse formation and maintenance in the hippocampus, but is also essential for excitatory synaptic transmission that requires the presence of Cbln2 (Tao et al., 2018). In the 5xFAD AD mouse model the CPTX fusion protein increased the frequency and amplitude of EPSCs and rescued long-term potentiation (LTP) in CA1 Schaffer collaterals (Suzuki et al., 2020). Interestingly, CPTX caused accumulation of AMPA receptors in the synapses of wild-type mice, but appeared to decrease AMPA receptor accumulation in 5xFAD mice. Additionally, recombinant Cbln1 mitigated hyperexcitability of amygdala neurons in rodent pain models by downregulating AMPA receptors (Narasimhan et al., 2025). A better understanding is needed about the role and regulation of Cbln1 on AMPA receptors and excitability at different stages of AD in preclinical models and human brains. These are crucial investigations to carry out because neuronal hyperexcitability and excitotoxicity may contribute to the neuronal stress at the root of AD pathogenesis and early AD propagation, whereas in late-stage AD this pattern inverts and hypo-excitability is observed (Targa Dias Anastacio et al., 2022; Liu et al., 2019; Decourt et al., 2022).

GluD1 is more abundant than GluD2 in the hippocampus (Nakamoto et al., 2020), and they exhibit overlapping functions with regard to synapse formation and stabilization (Lemoine et al., 2020; Ryu et al., 2012). This raises the question of whether GluD2 deficiency could cause biologically relevant pathology on its own, in the absence of GluD1 deficiency. While we were unable to find reports of targeted GluD2 knockout in the hippocampus, GluD2-specific effects in hippocampus are plausible. Specifically, GluD2 has recently been reported to exhibit ionotropic responses to D-serine and GABA under physiological temperatures (37 °C) but not at room temperature (22 °C). In contrast, GluD1 lacks similar response to these ligands at 37 °C (Vinnakota and Kumar, 2026). This difference suggests that loss of GluD2 in hippocampus could exert physiological effects even when normal levels of GluD1 are present.

Interestingly, we observed a biphasic change in GluD1, which showed a decrease in BB2 AD hippocampus that recovered in BB5/6 AD hippocampus. The reason for this is unclear, but it has been reported that GluD1 has an important role in hippocampal long-term depression via internalization of AMPA receptors via interactions with mGlu5 (Suryavanshi et al., 2016). We speculate that decreased GluD1 during early-stage AD would exacerbate hippocampal hyperexcitability while its increase in late-stage AD may be a compensatory response by postsynaptic neurons to counteract these changes.

Dysregulation of the autophagy pathway, which is known to be a player in AD pathology (Zhang et al., 2024), was associated with Cbln and GluD deficiency in our study. Our co-immunoprecipitation results suggest that transsynaptic complex machinery (GluD1) can interact with activators of autophagy (PIST). However, autophagy is a versatile cellular process triggered by many types of changes, and AD is a highly multi-factorial disease. Further work is needed to evaluate the degree to which autophagic downregulation in AD is driven by deficits in transsynaptic complex signaling versus other effectors of autophagy, and how autophagy dysregulation in turn affects synaptic function and neuroplasticity.

There are limitations of this work that should be noted. Firstly, because this work involved postmortem human tissues, it is not possible to determine whether the loss of transsynaptic complexes is a cause or a consequence of AD progression. Similarly, we could only identify molecular connections, but not causal relationships, between loss of transsynaptic complexes and altered markers of autophagy and neuroplasticity. Lastly, specimens from the GBB were only from female patients, and we only had three specimens from early-stage AD. A larger follow-up study will therefore be important to confirm our findings and determine whether they reflect AD progression in male patients. Nonetheless, the present study identified novel molecular differences in the AD brain that provide the rationale for mechanistic studies to better understand AD pathogenesis and early progression, as well as assess possible transsynaptic complex-based rescue paradigms for neurotrophic effects and to slow disease progression.

## Data Availability

The original contributions presented in the study are included in the article/Supplementary material, further inquiries can be directed to the corresponding author.
