# Supplementary material for: Transsynaptic complex dysfunction in the hippocampus of Alzheimer’s disease patients
Source: Front Aging Neurosci. 2026 Jun 10;18:1837327. doi: 10.3389/fnagi.2026.1837327 (PMC13290867; doi:10.3389/fnagi.2026.1837327)
Supplement: Supplementary file 1 [file Data_Sheet_1.docx]

Supplementary Material

| **Table S1.** Demographics, pathology, and RIN of specimens from the Garrison Brain Bank (GBB). | | | | | |
| --- | --- | --- | --- | --- | --- |
| **Investigational Group** | **Diagnosis** | **Sex** | **Age at Death (years)** | **B&B Stage** | **Average RIN Score** |
| Control | Control | F | 73 | 0 | 4.6 |
| Control | Control | F | 68 | 1 | 5.0 |
| Control | Control | F | 74 | 1 | 4.3 |
| Control | Control | F | 77 | 1 | 5.2 |
| Control | Control | F | 89 | 0 | 4.3 |
| Early AD | AD | F | 80 | 2 | 5.6 |
| Early AD | AD | F | 91 | 2 | 4.2 |
| Early AD | AD | F | 78 | 2 | 4.1 |
| Advanced AD | AD | F | 87 | 6 | 5.6 |
| Advanced AD | AD | F | 89 | 6 | 5.6 |
| Advanced AD | AD | F | 92 | 6 | 4.1 |
| Advanced AD | AD | F | 96 | 5/6 | 4.9 |
| Advanced AD | AD | F | 88 | 6 | 4.7 |
| Advanced AD | AD | F | 81 | 5 | 4.4 |
|  |  |  |  |  |  |

**
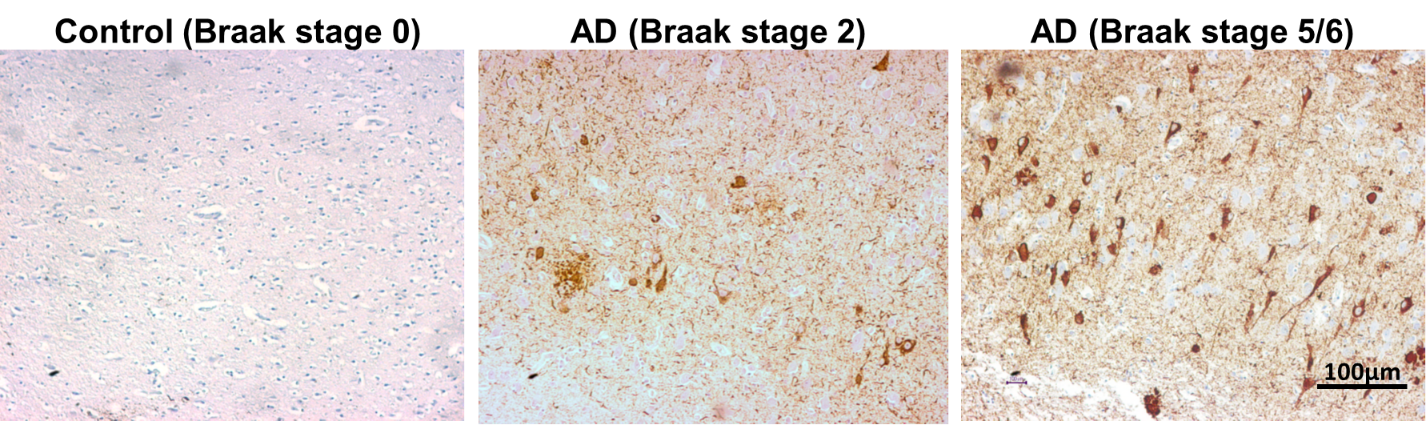
 Figure S1.** Braak staging of GBB samples was performed by licensed neuropathologists. Shown are examples of human hippocampal tissues stained for phospho-tau using the AT8 antibody, which is reactive against tau phosphorylated at serine 202 and threonine 205.

| **Table S2.** Demographics and pathology of specimens from the NIH NeuroBioBank. | | | | | |
| --- | --- | --- | --- | --- | --- |
| **Investigational Group** | **Diagnosis** | **Sex** | **Age at Death (years)** | **B&B Stage** | **Mean Aβ Plaque Area (mm²)** |
| Control | Control | F | 90 | 1 | 0 |
| Control | Control | F | 84 | 1 | 0 |
| Control | Control | F | 84 | 1 | 0 |
| Control | Control | F | 66 | 1 | 0 |
| Control | Control | F | 85 | 1 | 1.94 |
| Early AD | AD | M | 88 | 2 | 10.6 |
| Early AD | AD | F | 104 | 2 | 2.88 |
| Early AD | AD | F | 91 | 2 | 2.96 |
| Early AD | AD | M | 85 | 2 | 3.72 |
| Early AD | AD | F | 85 | 2 | 4.08 |
| Early AD | AD | M | 83 | 2 | 4.8 |
| Early AD | AD | F | 93 | 2 | 4.9 |
| Early AD | AD | F | 97 | 2 | 4.96 |
| Early AD | AD | M | 66 | 2 | 5.28 |
| Advanced AD | AD | M | 72 | 6 | 12.03 |
| Advanced AD | AD | M | 62 | 6 | 24.69 |
| Advanced AD | AD | M | 64 | 6 | 27.21 |
| Advanced AD | AD | F | 88 | 6 | 4.18 |
| Advanced AD | AD | M | 92 | 6 | 5.65 |
| Advanced AD | AD | F | 92 | 6 | 9.17 |
| Advanced AD | AD | F | 103 | 6 | 7.07 |
| Advanced AD | AD | F | 83 | 6 | 7.24 |
| Advanced AD | AD | M | 78 | 6 | 10.85 |

| **Table S3.** Primer sequences used for qPCR. | | | |
| --- | --- | --- | --- |
| **Gene** | **Forward primer (5'->3')** | **Reverse primer (5'->3')** | **Amplicon size** |
| *ACTB* | GGGCATGGGTCAGAAGGATT | TCGATGGGGTACTTCAGGGT | 81 |
| *CBLN1* | TGATTTCAGCCTTCGCTGGT | TATGCTCGGTCGCCTTTCTC | 91 |
| *CBLN2* | CTGCTGCTCATGGAAAGGGA | GTGGAGTATTTCCAGCCCCC | 80 |
| *GRID1* | CAGATTTCCCCAGCGTCGAT | GGTGTTCTGGTACTCCCGTG | 96 |
| *GRID2* | CACTGCGGTTGGTGACCTTA | GCTTGGAAAGGGTTGTTGCC | 99 |
| *NLGN1* | AATACCAGACGACCCCCAGA | CCTTCCCCTTGGTTCACTCC | 84 |
| *NRXN1* | CGAGCTCAGGTGGGTTAGC | TGGACTCCCGGATCACTTCT | 84 |
| *NRXN2* | ATCAACCGCATGCCCTTCC | GGCCACGTGTAGGTGATGAG | 92 |
| *SPARCL1* (hevin) | ATCCTGTGCACTGGCAGTTT | CAGAGATGCTCGCAGAGGAG | 92 |
